# Prognostic value of depression and anxiety on breast cancer recurrence and mortality: a systematic review and meta-analysis of 282,203 patients

**DOI:** 10.1038/s41380-020-00865-6

**Published:** 2020-08-20

**Authors:** Xuan Wang, Neng Wang, Lidan Zhong, Shengqi Wang, Yifeng Zheng, Bowen Yang, Juping Zhang, Yi Lin, Zhiyu Wang

**Affiliations:** 1 Integrative Research Laboratory of Breast Cancer, the Research Center for Integrative Cancer Medicine, Discipline of Integrated Chinese and Western Medicine & the Second Clinical College of Guangzhou University of Chinese Medicine, Guangzhou, Guangdong China; 2 Guangdong Provincial Key Laboratory of Clinical Research on Traditional Chinese Medicine Syndrome, Guangdong Provincial Academy of Chinese Medical Sciences, Guangdong Provincial Hospital of Chinese Medicine, Guangzhou,, 510006 Guangdong China; 3 College of Basic Medicine, Guangzhou University of Chinese Medicine, Guangzhou, Guangdong China; 4 School of Chinese Medicine, Hong Kong Baptist University, Hong Kong, China

**Keywords:** Depression, Psychology

## Abstract

Depression and anxiety are common comorbidities in breast cancer patients. Whether depression and anxiety are associated with breast cancer progression or mortality is unclear. Herein, based on a systematic literature search, 17 eligible studies involving 282,203 breast cancer patients were included. The results showed that depression was associated with cancer recurrence [1.24 (1.07, 1.43)], all-cause mortality [1.30 (1.23, 1.36)], and cancer-specific mortality [1.29 (1.11, 1.49)]. However, anxiety was associated with recurrence [1.17 (1.02, 1.34)] and all-cause mortality [1.13 (1.07, 1.19)] but not with cancer-specific mortality [1.05 (0.82, 1.35)]. Comorbidity of depression and anxiety is associated with all-cause mortality [1.34 (1.24, 1.45)] and cancer-specific mortality [1.45 (1.11, 1.90)]. Subgroup analyses demonstrated that clinically diagnosed depression and anxiety, being female and of younger age (<60 years), and shorter follow-up duration (≤5 years) were related to a poorer prognosis. Our study highlights the critical role of depression/anxiety as an independent factor in predicting breast cancer recurrence and survival. Further research should focus on a favorable strategy that works best to improve outcomes among breast cancer patients with mental disorders.

## Introduction

Breast cancer is one of the most prevalent cancers as well as the leading cause of death among women. According to the WHO statistics from 2018, breast cancer has an incidence of 11.6% among all types of cancer, accounting for 6.5% of mortalities worldwide [1]. The survival rate of breast cancer patients in developed countries has improved in recent decades due to the advances in early screening and treatment [2]. However, the mortality rate of breast cancer patients in some developing countries remains high due to inefficient screening and treatment, leaving an overall high death toll worldwide [3]. Recurrence and metastasis are the main causes of death in breast cancer. In addition, cardiovascular complications in breast cancer survivors were identified as another important cause of death among these women [4, 5]. Therefore, in order to improve the prognosis and overall survival, it is important to identify the risk factors influencing breast cancer-related death.

A number of factors can affect breast cancer progression and mortality. Recent studies have shown that the status of lymph nodes, tumor size, and histological subtypes are important risk factors determining its clinical outcomes [6–8]. In addition, clinical studies have demonstrated that demographic characteristics and lifestyle factors, such as age, obesity, smoking, and estrogen application, are also significantly related to breast cancer onset and development [9–13]. Importantly, with the transformation from the traditional medical system to a biopsychosocial medical network, much more attention has been paid to the role of psychological factors in the etiology and prognosis of breast cancer.

The diagnosis and treatment of breast cancer are frequently accompanied by changes in physical status and function, unpleasant side effects, decline in quality of life, and impaired social relationships [14, 15]. Therefore, breast cancer patients always suffer from negative emotions under chronic psychological stress. Clinical studies have also suggested that patients with breast cancer suffer a much higher prevalence of mental disorders compared with the general population. Depression and anxiety are the two most common psychiatric symptoms. Results from previous meta-analyses revealed that the prevalence of depression and anxiety among breast cancer patients was up to 32.2% and 41.9%, respectively [16, 17]. Depression and anxiety can affect the physiological function, treatment compliance, psychological function, and quality of life of patients with breast cancer and may even be an important factor affecting the mortality of breast cancer patients. Although whether psychological factors predict breast cancer mortality has been widely investigated, the findings are not consistent. Seven studies reported that depression was associated with an increase in the risk of mortality [18–24]. Another six studies found no association between depression and mortality [25–30]. Previous meta-analyses presented evidence that depression was related to mortality [31, 32] but not to progression in cancer patients [32]. However, these meta-analyses were constrained by high heterogeneity based on mixed types of cancers. Furthermore, as breast cancer is a hormone-dependent neoplasm, its response to mental illness may be distinct from that of other cancers. Meanwhile, several studies have assessed whether improvements in depression are associated with survival benefits. Giese-Davis et al. [33] found that a decrease in depression under supportive-expressive group therapy (SEGT) predicted longer survival for women with breast cancer. A recent meta-analysis also found that SEGT and other supportive psychotherapeutic interventions did improve overall cancer survival [34], while Kissane et al. [35] found no evidence that SEGT prolongs survival. With regard to anxiety, its influence on breast cancer prognosis is still under dispute. Shim et al. [24] found that anxiety is predictive of all-cause mortality for breast cancer patients. Groenvold et al. [27] demonstrated that anxiety was associated with relapse-free survival, but not with overall survival. However, several studies indicated that no association was found between anxiety and mortality in breast cancer patients [21, 23, 25, 28, 36]. Notably, animal models have clearly shown that chronic psychological stress can promote tumor growth and lung metastasis [37, 38]. Thaker et al. [39] found that chronic stress promotes ovarian tumor growth and angiogenesis in a murine model, and that adrenergic blockade reverses the effect. Hence, it is necessary to conduct comprehensive and rigorous studies to determine the relationship between depression and anxiety and breast cancer survival and progression.

Herein, we conducted a meta-analysis of depression and anxiety in relation to mortality and progression in patients with breast cancer. We collected and quantitatively integrated the data regarding the association between recurrence, all-cause mortality and cancer-specific mortality with depression and anxiety, respectively. The results demonstrate that depression and anxiety are both associated with recurrence and all-cause mortality. Furthermore, depression and comorbid depression and anxiety are related to cancer-specific mortality, while anxiety alone is not. Our findings highlight the importance of depression and anxiety in predicting breast cancer prognosis and suggest that careful consideration should be given to routine screening for mental distress in breast cancer patients.

## Methods

### Protocol and guidance

This study was performed in accordance with Preferred Reporting Items for Systematic Reviews and Meta-Analysis. The protocol for this review has been registered at PROSPERO (CRD42020162466).

### Search strategy

Following recommendations of the Meta-analysis of Observational Studies in Epidemiology group [40], we searched the following electronic databases for studies written in English from their inception until December 15, 2019: PubMed, Embase, The Cochrane Library, and PsycINFO. The search strategy was implemented using combined index terms (Medical Subject Headings, Emtree) and free-text keywords. Keywords included (“breast cancer” OR “breast neoplasm”) AND (“depression” OR “anxiety”) AND (“survival” OR “mortality” OR “metastasis” OR “recurrence”). Tables S1–S4 in the supplementary show the detailed methods used for searching all of the databases. Additional studies were searched by examining the reference lists of reviews and eligible publications for potentially eligible studies.

### Inclusion and exclusion criteria

The retrieved studies were eligible for qualitative and quantitative analysis if they (1) were cohort studies, (2) investigated patients with breast cancer, (3) assessed depression or anxiety by standard diagnostic criteria or self-report scales, and (4) provided HRs or risk ratios (RRs) and their 95% CIs for all-cause mortality or cancer-specific mortality.

The studies were excluded if they (1) had participants with malignancy other than breast cancer, (2) only recruited patients with breast cancer but without depression or anxiety, and (3) lacked measurement of cancer recurrence or mortality. In addition, if duplicate articles were derived from an identical or overlapping patient population, only the latest and/or complete one was used in the meta-analysis. When there were multiple groups of useful data in the same article, only the data derived from the group with the largest sample size were selected for the analysis.

### Data extraction and quality assessment

Two reviewers independently extracted the data from the included studies. The following details are presented in this review: first author’s name, year of publication, country, participant number, age, time of assessment of depression, follow-up duration, depression/anxiety measurement, and adjusted major confounders.

All of the selected articles were examined in terms of quality based on the Newcastle–Ottawa Quality Assessment Scale for cohort studies [41]. This semiquantitative scale uses a star system to assess the quality for eight items across three domains: selection (four items, one star each), comparability (one item, up to two stars), and exposure (three items, one star each). In this meta-analysis, we graded quality as good (≥7 stars), fair (4–6 stars), or poor (<4 stars). Any discrepancy between the two reviewers was resolved by discussion with the third reviewer.

### Statistical analysis

The outcomes were recurrence-free survival (the time until breast cancer recurrence, other malignant disease, or death), all-cause mortality (death from any cause), and breast cancer-specific mortality (death resulting from breast cancer) in patients with breast cancer based on the effects of depression and anxiety. The method was based on the HRs with 95% CIs obtained from each study. The relative risk (RR) was deemed approximatively equivalent to the HR if the HR was not reported. Most HRs were adjusted by different variables, such as age or tumor stage. If there were unadjusted HRs (or RRs) and adjusted ones, the adjusted were preferred. Subgroup analyses were conducted according to age (average ≤60 vs. >60 years), follow-up duration (<5 vs. ≥5 years), time of assessment of depression (before cancer diagnoses vs. after cancer diagnoses), and measure of mental status (clinical diagnose vs. symptom scale).

The Cochrane Q test and *I*^2^ statistic were employed to evaluate heterogeneity between studies, and an *I*^2^ > 50% was considered statistically significant [42]. If the *I*^2^ value was 50% or greater, we pooled the results using a random-effects model. Otherwise, a fixed-effect model was applied. The funnel plots and Egger test were used to detect potential publication bias [43, 44]. To assess the stability of the results, a leave-one-out sensitivity analysis was carried out. Analyses were performed with Stata 15.0 (StataCorp LP, College Station, TX, USA).

## Results

### Eligible studies and study characteristics

**Fig. 1:**
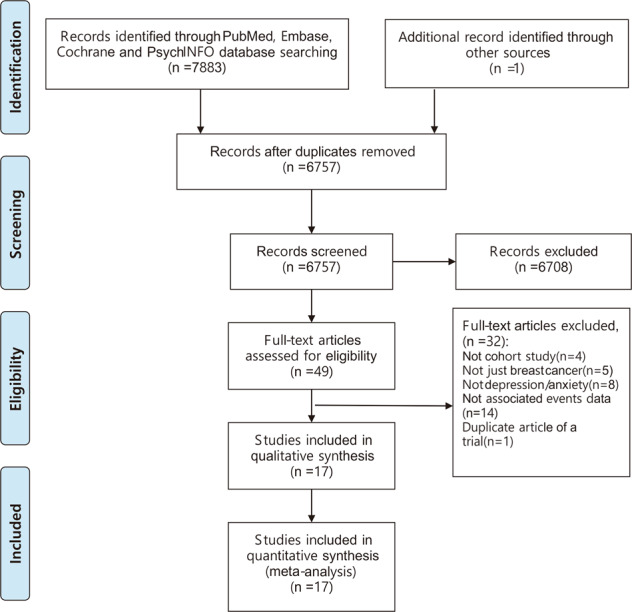
Flow chart of identification of eligible studies.

After identifying 7884 references, 1127 duplicate publications, and 6708 irrelevant studies were excluded, leaving 49 potentially eligible studies (Fig. 1). Finally, 17 cohort studies conducted between 2002 and 2019 were included in the meta-analysis.

The general characteristics of the included studies are listed in Table 1. A total of 282,203 patients with breast cancer were involved, and trials ranged in size from 34 to 124,381 participants. The duration of follow-up ranged from 2 to 25 years. Among these studies, nine studies were from Europe, five were from the US, two were from Asia, and one was from Australia. Mental symptoms were assessed by various tools, such as the Hospital Anxiety and Depression Scale (HADS), Center for Epidemiologic Studies Depression Scale (CES-D), Beck Depression Inventory (BDI), and 12-item General Health Questionnaire (GHQ-12), while clinical mental disorders were diagnosed based on the International Classification of Diseases (ICD) or the Diagnostic and Statistical Manual of Mental Disorders (DSM-MD). Most studies were adjusted for variables that affect the risk of cancer mortality, such as age, tumor stage, and comorbidities.

**Table 1:** Characteristics of Included Trials.

First Author (publication year), country	Number of participants	Age/years (mean)	Mental stress before/after cancer diagnosis	Follow-up duration/years (median)	Tool	Adjusted major confounders	Quality assessment
Graham (2002), the UK	202	48.4	Before/after	5	DSM-MD-3	Lymph node infiltration and histological type	8
Hjerl (2003), Denmark	20,593	20–70	Before/after	5.5 (before)/3.8 (after)	ICD-8	Age, histopathological grade, axillary lymph nodes removed, medical treatment period, and menopausal state	8
Goodwin (2004), the US	24,696	75	Before	3	ICD-9-CM	Age, ethnicity, comorbidity, American Joint Committee on Cancer Stage, and SEER site	6
Watson (2005), the UK	578	55	After	11.3	HADS	Age, histopathological grade, number of positive lymph nodes, pathological tumor size, type of surgery, treatment with radiotherapy, chemotherapy and/or endocrine therapy, and ER status.	9
Onitilo (2006), the US	912	72	After	8	CES-D	Age, race/ethnicity, poverty/income ratio, education, marital status, smoking, physical activity, BMI, aspirin use, and comorbid conditions	9
Groenvold (2007), Denmark	1588	52.4	After	12.9	HADS	Age, menopausal status, tumor size, histopathological diagnosis, tumor receptor status, grading of anaplasia, number of tumor-positive axillary nodes, adjuvant treatment regimen, type of operation, and local radiotherapy	9
Phillips (2008), Australian	708	<60	After	8.2	HADS	Grade, ER and PR status, size, number of involved nodes, body mass index, time to diagnosis from last childbirth, systemic treatment.	9
Bredal (2011), Norway	165	56.5	After	8.2	HADS	Age	8
Vodermaier (2014), the UK	1646	56.5	After	6.3	PSSCAN	Age	7
Chen (2016), China	3441	51	After	2.5	ICD-9-CM	Age, outpatient visits, adjuvant therapies, all comorbidities, CCI.	7
Kanani (2016), the UK	955	≥30	Before/after	10	ICD-10	Age, ethnicity, deprivation, comorbidities, stage and recorded cancer treatment	8
Eskelinen (2017), Finland	34	51.6	Before	25	BDI	–	6
Iglay (2017), the US	19,028	≥68	Before	5	ICD-9-CM	Age, income, race, ethnicity, SEER location, and marital status.	7
Liang (2017), the US	3095	63.1	Before	6.8	CES-D	Age, race, body mass index, smoking, postmenopausal hormone therapy, comorbidity, mammography use, tumor stage, tumor grade, and ER and PR status.	9
Desai (2019), the US	1474	–	Before	2	ICD-9-CM	County-level information, individual sociodemographic information, cancer stage, comorbidities, substance abuse, pain condition, and index year.	7
Shim (2019), Korea	124,381	50.2	After	4.2	ICD-10	Age, sex, place, income, CCI, disability, type of breast cancer, chemotherapy, radiation therapy, hormonal therapy, and target therapy	7
Batty (2017), the UK	78,707	54.9	After	9.5	GHQ-12	Age, BMI, educational attainment, smoking status, and frequency of alcohol consumption	9

*DSM-MD-3* the Diagnostic and Statistical Manual of Mental Disorders (third edition, revised), *HADS* hospital anxiety and depression scale, *BDI* Beck Depression Inventory, *PSSCAN* the 21-item psychosocial screen for cancer, *ICD* International Classification of Diseases, *CES-D* Center for Epidemiologic Studies Depression Scale, *SEER* Surveillance Epidemiology and End Results, *CCI* Charlson Comorbidity Index, *ER* estrogen receptor, *PR* progesterone receptor.

According to quality assessment criteria, 15 studies were graded as good quality and two studies as fair quality. Detailed information is listed in Supplementary Table S5.

### Effect of depression on recurrence and mortality in breast cancer patients

**Fig. 2:**
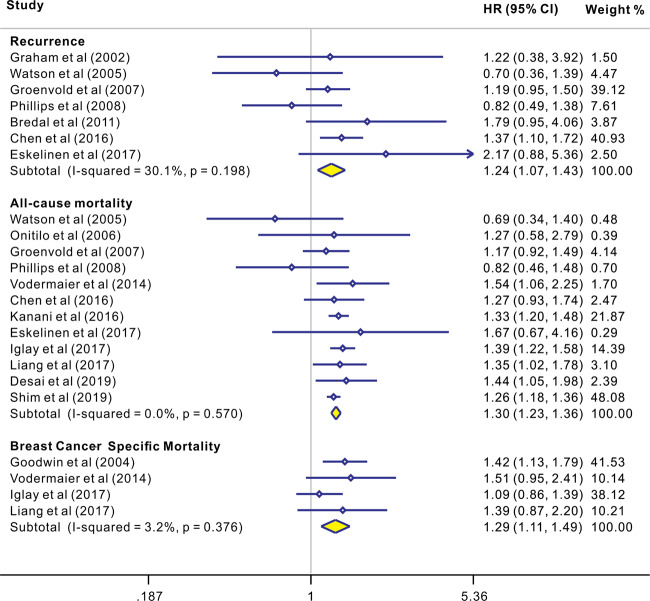
The effects of depression on recurrence, all-cause mortality, and cancer-specific mortality in patients with breast cancer. Results of individual and summary HR estimates, 95% CI, and weights of each study were shown. Diamonds indicate study specific HRs; Horizontal lines represent 95% CI; Arrowheads indicate error bars that extend beyond the area shown.

As shown in Fig. 2, seven studies [25, 27–30, 36, 45] yielded a pooled HR of 1.24 (1.07, 1.43) for the association between depression and recurrence based on fixed-effects model analyses. Low heterogeneity was observed, with *I*^2^ = 30.1% and *P* = 0.198. Furthermore, 12 studies [19–30] yielded a pooled HR of 1.30 (1.23, 1.36) for the association between depression and all-cause mortality. Low heterogeneity was observed, with *I*^2^ = 0.0% and *P* = 0.57. Finally, four studies [18, 19, 21, 22] yielded a pooled HR of 1.29 (1.11, 1.49) for the association between depression and breast cancer-specific mortality. Low heterogeneity was observed, with *I*^2^ = 3.2% and *P* = 0.376.

### Recurrence

**Fig. 3:**
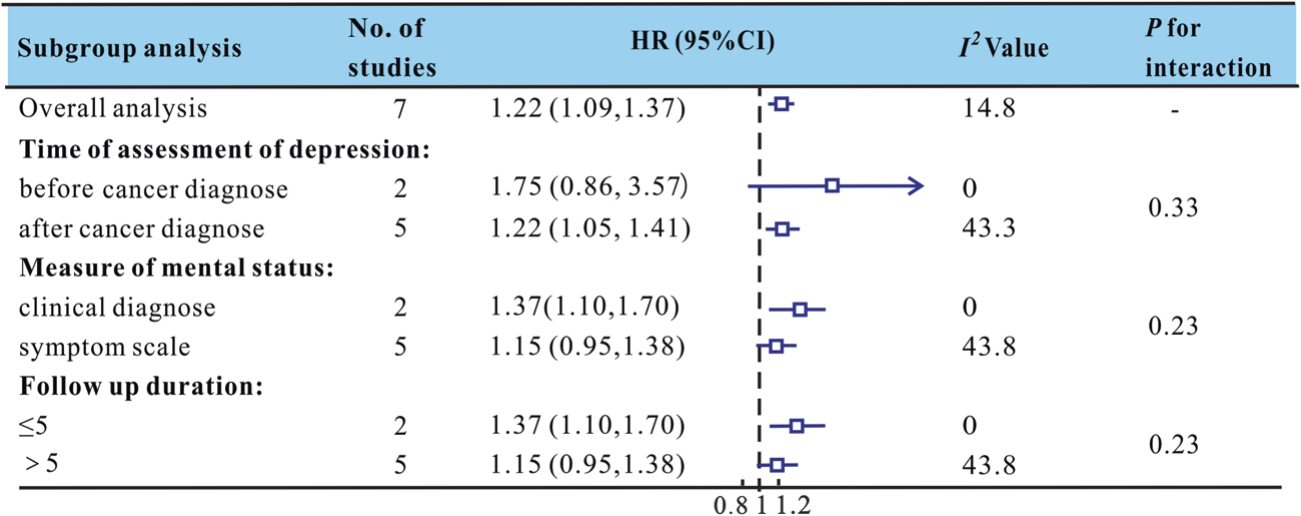
The effect of depression on recurrence: results of the subgroup analyses. Studies reported results stratified by time of assessment of depression, measure of mental status and follow up duration, respectively. Squares indicate study specific HRs; Horizontal lines represent 95% CI. Arrowheads indicate error bars that extend beyond the area shown.

The results showed that depression was associated with a 24% increase in the risk of cancer recurrence. In seven studies, the mean age of patients was <60 years. Two studies assessed depression before the breast cancer diagnosis, and five studies assessed it after the breast cancer diagnosis. Five studies assessed the effect of psychological symptoms using self-report scales, and two studies assessed it through clinical standardized interviews. There were two studies with a follow-up duration shorter than or equal to 5 years postdiagnosis and five studies with a follow-up duration longer than 5 years. Subgroup analysis, according to the assessment time of depression, mental status measurement tool, and follow-up duration, was performed (Fig. 3). The results showed that the prognostic impact of the depression was significant when depression was assessed after the breast cancer diagnosis [1.22 (1.05, 1.41)]. In addition, clinically diagnosed depression disorders were associated with recurrence [1.37 (1.10, 1.70)], while mental distress assessed by the symptom scale was not [1.15 (0.95, 1.38)]. In addition, studies with short-term follow-up (≤5 years) tended to report a higher recurrence risk [1.37 (1.10, 1.70)] compared with studies with longer follow-up of more than 5 years [1.15 (0.95, 1.38)].

### All-cause mortality

**Fig. 4:**
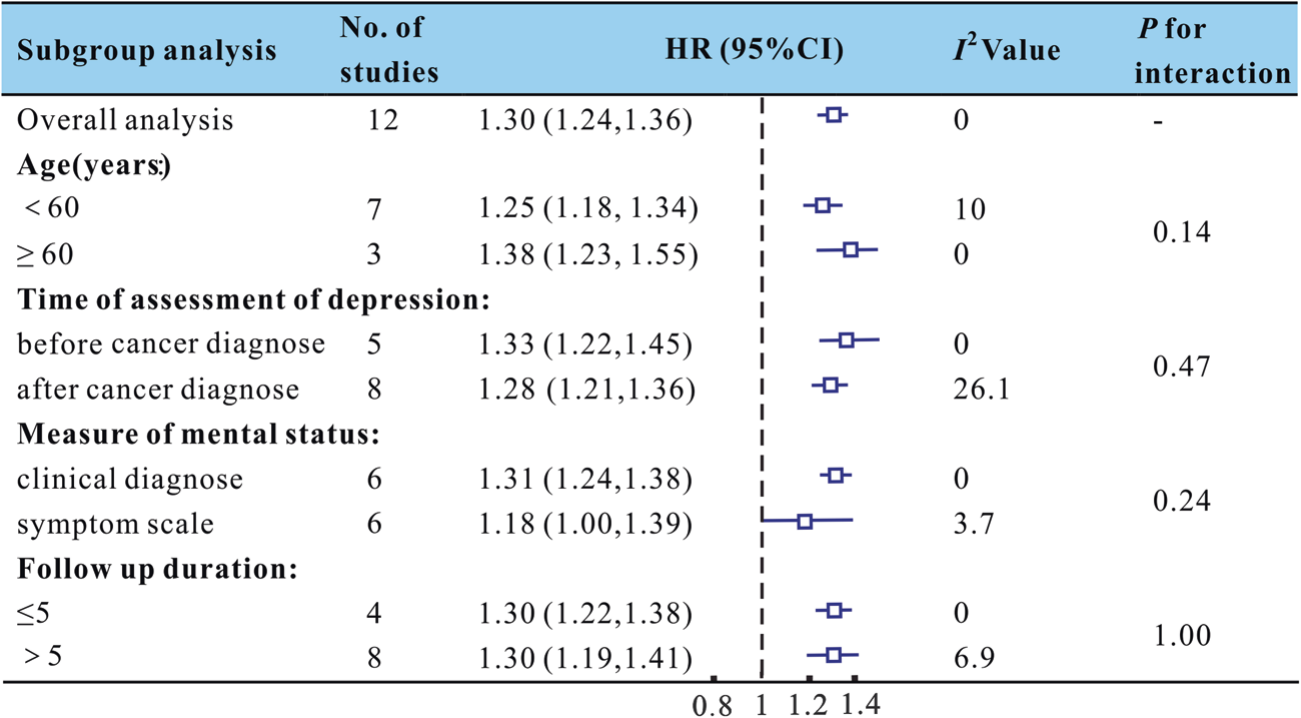
The effect of depression on all-cause mortality: results of the subgroup analyses. Studies reported results stratified by age, time of assessment of depression, measure of mental status and follow up duration, respectively. Squares indicate study specific HRs; Horizontal lines represent 95% CI.

The results showed that depression was associated with a 30% increase in the risk of all-cause mortality. The mean age of patients was <60 years in seven studies and over 60 years in three studies. Five studies assessed depression before breast cancer diagnosis, and eight studies assessed it after breast cancer diagnosis. Six studies assessed the effect of psychological symptoms using self-report scales, and six studies assessed it through clinical standardized interviews. There were four studies with follow-up duration ≤5 years postdiagnosis, and eight studies with follow-up duration longer than 5 years. Subgroup analysis was performed according to age, the assessment time of depression, the mental status measurement tool, and follow-up duration (Fig. 4). The results showed that the association was significant in both patients aged <60 years and those aged ≥60 years [1.25 (1.18, 1.34) and 1.38 (1.23, 1.55), respectively]. The significant prognostic impact of depression did not differ depending on whether it was assessed before [1.33 (1.22, 1.45)] or after [1.28 (1.21, 1.36)] the breast cancer diagnosis. Furthermore, there was no significant difference in risk between studies with a follow-up duration ≤5 years [1.30 (1.22, 1.38)] and those with a follow-up duration >5 years [1.30 (1.19, 1.41)]. However, clinically diagnosed depression disorders were associated with increased all-cause mortality [1.31 (1.24, 1.38)], while self-reported psychological symptoms were not [1.18 (1.00, 1.39)].

### Breast cancer-specific mortality

Likewise, the results showed that depression was associated with a 29% increase in the risk of breast cancer-specific mortality. The mean age of patients was <60 years in one study and over 60 years in three studies. Three studies assessed depression before the breast cancer diagnosis, and one study assessed it after the breast cancer diagnosis. One study assessed the effect of depressive symptoms using self-report scales, and three studies assessed it through clinical standardized interviews. There were two studies with a follow-up duration shorter than or equal to 5 years postdiagnosis and two studies with a follow-up duration longer than 5 years.

### Effect of anxiety on recurrence and mortality in breast cancer patients

**Fig. 5:**
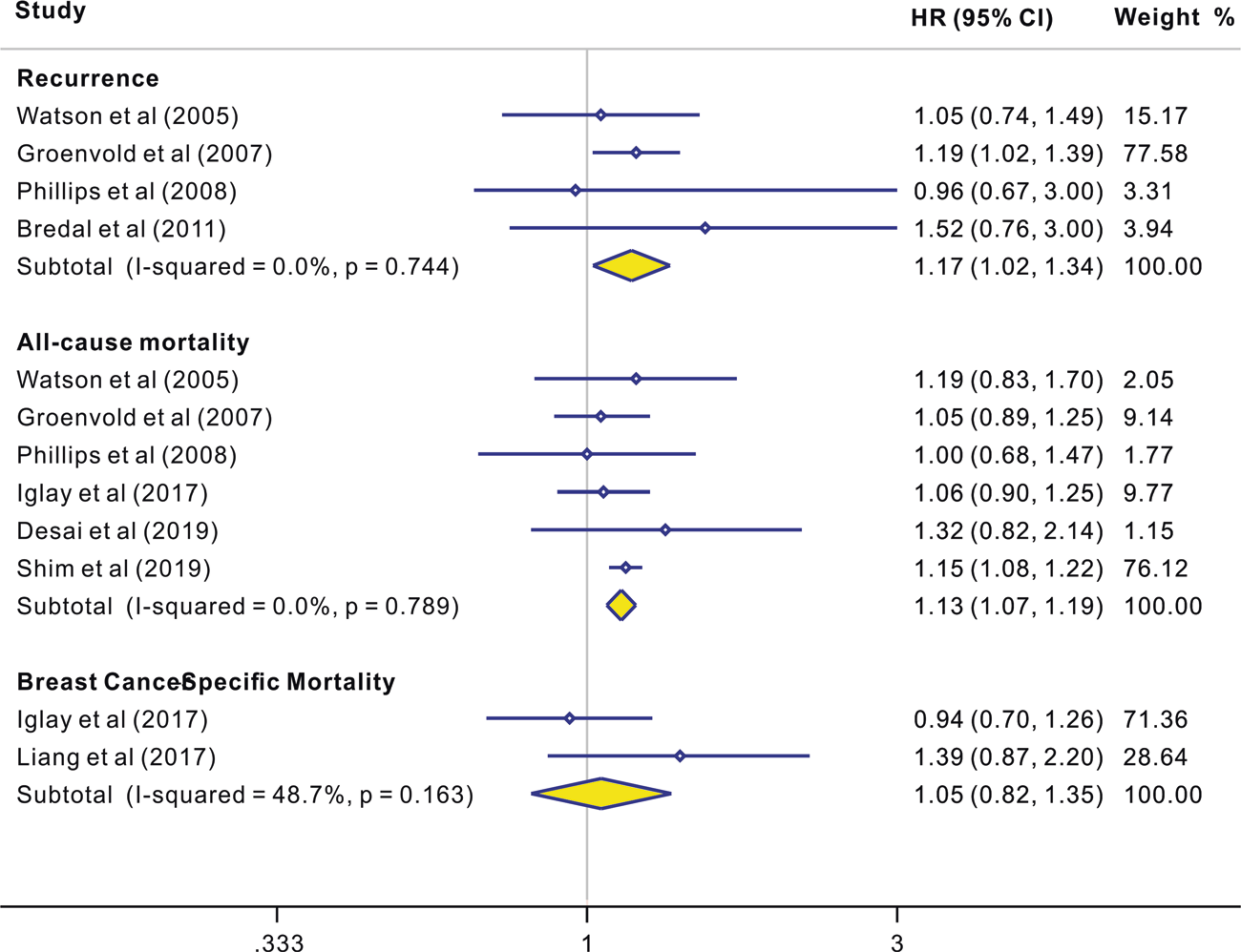
The effects of anxiety on recurrence, all-cause mortality, and cancer-specific mortality in patients with breast cancer. Results of individual and summary HR estimates, 95% CI, and weights of each study were shown. Diamonds indicate study specific HRs; Horizontal lines represent 95% CI.

As shown in Fig. 5, four studies [25, 27, 28, 36] yielded a pooled HR of 1.17 (1.02, 1.34) for the association between anxiety and recurrence based on fixed-effects model analyses. Low heterogeneity was observed, with *I*^2^ = 0.0% and *P* = 0.744. Furthermore, six studies [21, 23–25, 27, 28] yielded a pooled HR of 1.13 (1.07, 1.19) for the association between anxiety and all-cause mortality. Low heterogeneity was observed, with *I*^2^ = 0.0% and *P* = 0.789. However, there was no association between anxiety and breast cancer-specific mortality in two studies [1.05 (0.82, 1.35)] [21, 22]. Low heterogeneity was observed, with *I*^2^ = 48.7% and *P* = 0.163.

### Recurrence

The results showed that depression was associated with a 17% increase in the risk of cancer recurrence. In four studies, the mean age of patients was <60 years. All included studies measured anxious symptoms using the HAD scale after the breast cancer diagnosis, and four studies had a follow-up duration longer than 5 years. Thus, subgroup analysis could not be carried out.

### All-cause mortality

The results showed that anxiety was associated with a 13% increase in the risk of all-cause mortality. The mean age of patients was <60 years in four studies and over 60 years in one study. Two studies measured anxiety before the breast cancer diagnosis, and four studies measured it after the breast cancer diagnosis. Three studies assessed the effect of anxiety symptoms using self-report scales, and three studies assessed it through clinical standardized interviews. There were three studies with a follow-up duration shorter than or equal to 5 years postdiagnosis and three studies with a follow-up duration longer than 5 years. Subgroup analysis according to age, the assessment time of anxiety, the mental status measurement tool, and follow-up duration was performed (Supplementary Fig. S1). The association was significant only in several subgroups: patients aged <60 years [1.13 (1.07, 1.20)], anxiety assessed after breast cancer diagnosis [1.13 (1.07, 1.20)], clinically diagnosed anxiety disorders [1.14 (1.08, 1.20)], and follow-up duration ≤5 years [1.14 (1.08, 1.20)].

### Breast cancer-specific mortality

The results showed that anxiety was not related to the risk of breast cancer-specific mortality. The mean age of patients was over 60 years in two studies. Both studies measured depression before breast cancer diagnosis. One study assessed the effect of depressive symptoms using self-report scales, and the other study assessed it through clinical standardized interviews. One study had a follow-up duration ≤5 years postdiagnosis, and the other study had a follow-up duration longer than 5 years. Considering the limited studies included, subgroup analysis was not performed.

### Effect of depression and anxiety on mortality in breast cancer patients

As shown in Supplementary Fig. S2, four studies [21, 23, 24, 46] yielded a pooled HR of 1.34 (1.24, 1.45) for the association between depression and anxiety and all-cause mortality based on fixed-effects model analyses. Low heterogeneity was observed, with *I*^2^ = 0.0% and *P* = 0.755. Furthermore, two studies [21, 47] yielded a pooled HR of 1.45 (1.11, 1.90) for the association between depression and anxiety and breast cancer-specific mortality. Low heterogeneity was observed, with *I*^2^ = 49.3% and *P* = 0.160.

### Publication bias and sensitivity analysis

Due to the small number of studies for both depression and anxiety, funnel plots and Egger’s tests were only created/computed for the primary outcome of depression or anxiety. There was no evidence of publication bias based on visual inspection of funnel plots (Supplementary Fig. S3) or according to Egger’s tests (*P* > 0.1). Sensitivity analysis was performed to assess the stability of the results, and a single study from the meta-analysis was omitted each time. The results showed that the corresponding pooled estimates were not significantly altered, indicating that no individual study influences the results (Supplementary Fig. S4).

## Discussion

The meta-analysis confirms that depression, anxiety, and the combination of both are associated with increased recurrence and all-cause mortality in patients with breast cancer. In our study, depression predicted a 30% increase in mortality risk among cancer patients, which is more severe than the increased risk of 18% found by a study carried out in 2010 [31]. This may indicate an increasing tendency in the mortality risk in recent years. Importantly, in contrast to previous depression-focused meta-analyses, our findings show that anxiety can also predict recurrence and all-cause mortality in breast cancer patients. The all-cause mortality risk is the highest in patients with combined depression and anxiety, followed by those with depression alone. In addition, depression predicts a slightly higher risk of recurrence than anxiety. Depression and comorbid depression and anxiety can also increase the risk of cancer-specific mortality, while anxiety does not. Our results suggest that depression is a stronger risk factor for cancer progression and mortality than anxiety, and comorbid depression and anxiety are most closely related to mortality. Considering that depression and anxiety often co-occur clinically, more attention should be paid to them during the treatment of cancer patients.

There are several factors that may explain the increased mortality rate of breast cancer patients with depression and anxiety, such as lifestyle, behavioral factors, and biological factors. First, patients with mental disorders may be more prone to unhealthy lifestyle factors, such as smoking, drinking, obesity, and insomnia, which may increase the risk of mortality and cardiovascular disease [48]. Second, treatment nonadherence is also a severe problem. For instance, breast cancer patients may have difficulties adhering to chemotherapy and other complementary treatments [49], which could lead to poor prognosis. Furthermore, mortality could also be caused by nonnatural causes of death, especially suicide [50]. Depression and anxiety are also related to biological mechanisms such as abnormal activation of the hypothalamic–pituitary–adrenal axis and high norepinephrine and cortisol levels in patients [51, 52]. Thaker et al. [39] validated that chronic stress could lead to higher levels of tissue catecholamines, greater tumor burden, and more invasive growth of ovarian carcinoma. Bai et al. [53] reported that stress-induced epinephrine enhances lactate dehydrogenase A and promotes breast cancer stem-like cells. Kamiya et al. [54] found that chronic stress accelerates cancer growth and progression via stimulation of sympathetic neural nerves in tumor. It is interesting to investigate the expression profiles of neuroendocrine-related receptors in breast cancer and to develop novel targeting strategies in the future.

The different effects of depression and anxiety on breast cancer may indicate their distinct mechanisms. Anxiety is characterized by prominent tension, worry, and feelings of apprehension. Depression is indicated by depressive mood, slow thinking, and loss of interest. Patients with depression are more prone to committing suicide than those with anxiety, which may contribute to the higher all-cause mortality. Both depression and anxiety can lead to insomnia. However, anxiety is characterized by difficulty in falling asleep, and depression is characterized by waking up early. Different manifestations reflect different mechanisms of neuroendocrine disorders. More research is still needed to explore the distinct mechanisms between depression and anxiety.

The associations between depression/anxiety and mortality vary by age, assessment tools and time, and follow-up duration. Pinquart et al. [31] observed a stronger association between depression and mortality in older patients than in younger patients on the basis of the association between depression and various types of cancers. However, our study showed different results, namely that younger patients had a higher mortality risk compared with the elderly. Considering that our research is breast cancer-oriented and more representative for breast cancer, the inconsistent results may suggest that the mechanisms for mortality may differ across different types of cancer. It is believed that the severity of depression and anxiety is negatively correlated with age [55, 56]. In addition, younger patients with depression may have more difficulty in coping with cancer and may be more distressed due to their multiple social roles and heavier family burdens [19]. Therefore, younger women with breast cancer on average have a poorer prognosis than older ones.

Furthermore, we found that mental disorders diagnosed by a clinical interview were linked to a higher recurrence risk and poorer cancer survival than those assessed by self-rating scales. As patients with more severe depressive symptoms tend to be evaluated by clinical diagnostic interviews, whereas those with mild and moderate depressive symptoms are usually assessed by self-rating scales [57], this finding reflects the dose–response relationship to some extent—that is, the more severe the depression is, the higher the mortality becomes. In addition, depression assessment after cancer diagnosis was more strongly associated with worse survival than a prior diagnosis of depression. A prior diagnosis of depression may represent early detection, diagnosis, and treatment, which are key to improving the survival rate. This finding suggests that we should pay more attention to the early detection of patients’ psychological state and strive to enhance their mental health.

Moreover, the length of follow-up was related to different mortality risks. Studies with short-term follow-up tended to report higher mortality compared with studies with a longer follow-up of more than 5 years, consistent with the finding of Watson et al. [25, 58], that there is a significant association between depression and mortality at 5 years’ follow-up but not at 10 years. Another meta-analysis also showed that the association was the strongest in studies with intervals of ≤2 years [31]. This could be explained by the severity of depression and anxiety weakening over time [59], resulting in a decrease in the strength of the correlation between mental distress and mortality over time. Hence, more attention should be paid to the early period after cancer diagnosis. Interestingly, there is evidence that the symptom-management approach of cognitive-behavioral therapy may have more profound effects among recently diagnosed patients, while the emotionally expressive existential focus of SEGT may be more helpful in the later stages of progressive cancer. Thus, when choosing the psychosocial interventions, the phase of the disease should be considered [34].

Interestingly, most reported associations were adjusted by demographic and tumor-related variables, such as tumor stage, positive lymph nodes, and tumor size. Mental distress was still associated with poorer prognosis after adjustment for the variables, which suggests that it is an independent risk factor for cancer mortality. However, the strength of the correlation between mental distress and mortality was gradually attenuated after adjustment (Supplementary Table S6). This variation was also found in previous similar meta-analyses of the association between depression in cardiac patients and prognosis [57, 60]. One plausible explanation is that the association between breast cancer and mental distress is bidirectional—that is, the severity of the cancer in turn leads to a poorer prognosis and mental disorders.

Nevertheless, there are several limitations to the current meta-analysis. First, as previously discussed, histological subtypes, tumor stage, and the status of hormone receptors may affect the association between mental distress and cancer progression and mortality. However, because each study adjusted for a different set of variables, a pooled association consistently adjusted for the same variables across the studies could not be reached. Furthermore, as the majority of cases in this study had mixed pathological subtypes, tumor stages, and hormone receptors, subgroup analysis for further comparison based on these factors also could not be carried out. Second, we could not accurately compare the different levels of depression/anxiety in the included trials due to the various tools for assessing depression/anxiety used in the included trials. This is one of the reasons this study did not examine the dose–response associations between depression/anxiety and mortality. Third, misclassification could have occurred, as depression and anxiety were usually evaluated only at one time, while these mental disorders are not fixed and prone to fluctuations over time.

### Implications

Our findings strongly support the role of depression and anxiety in breast cancer progression and mortality, and they have important public health implications. First, more attention should be paid to psychosocial distress in breast cancer patients. Given the high prevalence of psychosocial distress and the resulting adverse effects on cancer recurrence and mortality, more health education should be carried out to make doctors, patients, and their families aware of the importance of psychological factors. Second, routine screening and early detection is recommended. As depression and anxiety may change across the trajectory of cancer care, screening at regular intervals can help effectively monitor psychological changes in the early period and allow for timely intervention to prevent it from getting worse. Furthermore, our study indirectly highlights the significance of treatment for depression and anxiety in breast cancer patients. It is believed that psychotherapy and mind–body therapies may relieve depressive symptoms in patients with breast cancer [61]. However, doctors should be careful when prescribing antidepressants. Several studies have reported that antidepressant use is associated with increased risk of cardiovascular disease mortality and all-cause mortality [62, 63], while others did not observe an association between antidepressant use and the risk of recurrence and mortality [64, 65]. Therefore, physicians, oncologists, and psychiatrists should work together to develop better options in the coordination of care and treatment for these patients.

## Conclusion

Our results indicate that depression and anxiety both have adverse effects on recurrence and all-cause mortality in patients with breast cancer, and depression can predict cancer-specific mortality, while anxiety cannot. Comorbidity of depression and anxiety is associated with all-cause mortality and cancer-specific mortality. The findings indirectly support the need for early and regular detection and timely treatment of mental disorders in patients with breast cancer, especially in the early period after cancer diagnosis. Furthermore, physicians should work more closely with psychiatrists and oncologists on the coordination of care and treatment for these patients. However, further research is required to identify the intervention strategies and coordination models that work best to improve outcomes among cancer patients with mental disorders and to further clarify the causal pathways between mental illness and worse survival.

## Supplementary information

Supplemental material
