# Supplementary material for: Prognostic value of depression and anxiety on breast cancer recurrence and mortality: a systematic review and meta-analysis of 282,203 patients
Source: Mol Psychiatry. 2020 Aug 20;25(12):3186–97. doi: 10.1038/s41380-020-00865-6 (PMC7714689; doi:10.1038/s41380-020-00865-6)
Supplement: Supplementary file 1 — Supplemental material [file 41380_2020_865_MOESM1_ESM.doc]

**Data Supplement**

**Prognostic value of depression and anxiety on breast cancer recurrence and mortality:**

**A systematic review and meta-analysis of 282, 203 patients**

| Contents |  | Pages |
| --- | --- | --- |
| Supplementary Table S1 | Search strategy for Pubmed | 2 |
| Supplementary Table S2 | Search strategy for Embase | 4 |
| Supplementary Table S3 | Search strategy for Cohorane | 5 |
| Supplementary Table S4 | Search strategy for PsycINFO | 6 |
| Supplementary Table S5 | Quality assessment according to NOS | 7 |
| Supplementary Figure S1 | Subgroup analysis of the eﬀect of anxiety on all-cause mortality | 8 |
| Supplementary Figure S2 | Forest plot illustrating the effects of depression and anxiety on all-cause mortality and cancer-specific mortality in patients with breast cancer | 8 |
| Supplementary Figure S3 | Funnel plots for the assessment of publication bias | 9 |
| Supplementary Figure S4 | Sensitivity analysis | 9 |
| Supplementary Table S6 | Comparison between crude and adjusted association | 10 |
|  |  |  |

**Supplementary Table S1. Search strategy for Pubmed**

| #1 | Search ((((((((breast Neoplasm) OR "Breast Neoplasms"[Mesh])) OR Neoplasm*,Breast[Title/Abstract]) OR Breast Tumor*[Title/Abstract]) OR Tumor*, Breast[Title/Abstract]) OR Breast Cancer[Title/Abstract]) OR Cancer, Breast[Title/Abstract]) OR Mammary Cancer*[Title/Abstract]) OR Cancer*, Mammary[Title/Abstract]) OR Malignant Neoplasm of Breast[Title/Abstract]) OR Breast Malignant Neoplasm*[Title/Abstract]) OR Malignant Tumor of Breast[Title/Abstract]) OR Breast Malignant Tumor*[Title/Abstract]) OR Cancer of Breast[Title/Abstract]) OR Cancer of the Breast[Title/Abstract]) OR Mammary Carcinoma*, Human[Title/Abstract]) OR Carcinoma*, Human Mammary[Title/Abstract]) OR Human Mammary Carcinoma*[Title/Abstract]) OR Mammary Neoplasm*, Human[Title/Abstract]) OR Human Mammary Neoplasm*[Title/Abstract]) OR Neoplasm*, Human Mammary[Title/Abstract]) OR Breast Carcinoma*[Title/Abstract]) OR Carcinoma*, Breast[Title/Abstract] | 403649 |
| --- | --- | --- |
| #2 | Search ((depress*[Title/Abstract]) OR ((((ressivel Disorder*[Title/Abstract]) OR Disorder*, Depressive[Title/Abstract]) OR Neuros*, Depressive[Title/Abstract]) OR Depressive Neuros* [Title/Abstract]) OR Depression*, Endogenous[Title/Abstract]) OR Endogenous Depression* [Title/Abstract]) OR Depressive Syndrome*[Title/Abstract]) OR Syndrome*, Depressive[Title/Abstract]) OR Depression*, Neurotic[Title/Abstract]) OR Neurotic Depression*[Title/Abstract]) OR Melancholia* [Title/Abstract]) OR Unipolar Depression*[Title/Abstract]) OR Depression*, Unipolar[Title/Abstract])) OR "Depressive Disorder"[Mesh]) OR ((((Anxiety'lMesh]) OR Hypervigilance[Title/Abstract]) OR Nervousness[Title/Abstract]) OR Social Anxiet*[Title/Abstract]) OR Anxiet*, Social[Title/Abstract]) OR Depressive Symptom*[Title/Abstract]) OR Symptom*, Depressive[Title/Abstract]) OR Emotional Depression*[Title/Abstract]) OR Depression*, Emotional[Title/Abstract]) OR ((Depression) OR "Depression'"[Mesh])) | 597366 |
| #3 | Search (((Rcurrence*[Title/Abstract]) OR Recrudescence*[Title/Abstract]) OR Relapse*[Title/Abstract)) OR "Recurrence"[Mesh]) OR (((Metastases, Neoplasm[Title/Abstract]) OR Neoplasm Metastases[Title/Abstract]) OR Metastasis[Title/Abstract]) OR Metastases[Title/Abstract]) OR Metastasis, Neoplasm[Title/Abstract])) OR "Neoplasm Metastasis"[Mesh]) OR "Death"[Mesh]) OR "Survival"[Mesh]) OR (((((((Mortalit*[TitlelAstract]) OR Case Fatality Rate*[Title/Abstract]) OR Rate*, Case Fatality[Title/Abstract]) OR Mortalit*, Excess[Title/Abstract]) OR Excess Mortalit* [Title/Abstract]) OR Decline*, Mortality[Title/Abstract]) OR Mortality Decline*[Title/Abstract]) OR Mortality Determinant*[Title/Abstract]) OR Determinant*, Mortality[Title/Abstract]) OR Mortalit*, Differential[Title/Abstract]) OR Differential Mortalit*[Title/Abstract]) OR Age*Specific Death Rate* [Title/Abstract]) OR Death Rate*, Age-Specific[Title/Abstract]) OR Rate*, Age-Specific Death[Title/Abstract]) OR Death Rate*[Title/Abstract]) OR Rate*, Death[Title/Abstract]) OR Mortality Rate*[Title/Abstract]) OR Rate*, Mortality[Title/Abstract])) OR "Mortality"[Mesh] | 1981962 |
| #4 | #1 AND #2 AND #3 | 862 |
| #5 | #4 Filters: Humans | 570 |

**Supplementary Table S2**. **Search strategy for Embase**

| #1 | 'breast cancer':ti,ab ,kw | 386,218 |
| --- | --- | --- |
| #2 | 'breast cancer'/exp | 456,848 |
| #3 | #1 OR #2 | 533,696 |
| #4 | 'depression'/exp OR depression:ti,ab,kw | 670,517 |
| #5 | 'depressive disorder*/exp OR 'depressive disorder*:ti,ab ,kw | 483,174 |
| #6 | 'anxiety'/exp OR anxiety:ti,ab,kw | 322,685 |
| #7 | #4OR#5OR#6. | 834,022 |
| #8 | 'survival'/exp OR survival:ti,ab,kw | 1,596,312 |
| #9 | 'mortality'/exp OR mortality:ti,ab,kw | 1,436,301 |
| #10 | 'metastasis'/exp OR metastasis:ti,ab,kw | 711,312 |
| #11 | 'recurrent disease'/exp OR 'recurrent disease':ti,ab kw OR recurrence:ti,ab,kw | 547,522 |
| #12 | #8 OR #9 OR #10 OR #11 | 3,497,669 |
| #13 | #3 AND #7 AND #12 | 3,547 |
| #14 | #13 AND 'human'/de | 1,529 |

**Supplementary Table S3**. **Search strategy for Cochrane**

| #1 | MeSH descriptor: [Breast Neoplasms] | 11950 |
| --- | --- | --- |
| #2 | MeSH descriptor: [Depression] | 10762 |
| #3 | MeSH descriptor: [Anxiety Disorders] this term only | 0 |
| #4 | MeSH descriptor: [Anxiety] | 7073 |
| #5 | MeSH descriptor: [Survival] | 130 |
| #6 | MeSH descriptor: [Mortality] | 511 |
| #7 | MeSH descriptor: [Neoplasm Metastasis] | 2894 |
| #8 | MeSH descriptor: [Recurrence] | 11704 |
| #9 | (breast cancer);ti,ab,kw | 35107 |
| #10 | (depression);ti,ab,kw | 79844 |
| #11 | (Depressive Disorder):ti,ab,kw | 34234 |
| #12 | (anxiety Disorder):ti,ab,kw | 0 |
| #13 | (anxiety)ti,ab,kw | 46561 |
| #14 | (survival):ti,ab,kw | 104831 |
| #15 | (mortality):ti,ab,kW | 88849 |
| #16 | (metastasis);ti,ab,kw | 20044 |
| #17 | (recurrence)ti,ab,kw | 67697 |
| #15 | #1 or #9 | 36060 |
| #16 | #5 or #6 or #7 or #8 or #13 or#14 or #15 or #16 or #17 | 212737 |
| #17 | #2 or #3 or #4 or #11 or #12 or #13 | 525204 |
| #18 | #15 and #16 and #17 in Trials | 5264 |

**Supplementary Table S4. Search strategy for PsycINFO**

| 1 | exp Breast Neoplasms/ or breast cancer.mp. | 12350 |
| --- | --- | --- |
| 2 | exp "Depression (Emotion)"/ or depression.mp. | 293971 |
| 3 | exp Anxiety Disorders/ or exp Major Depression/ or exp Bipolar Disorder/ or Depressive disorder.mp | 238394 |
| 4 | exp Anxiety/ or anxiety.mp. | 196750 |
| 5 | survival.mp. | 37035 |
| 6 | morality.mp. or exp "Death and Dying"/ | 57290 |
| 7 | metastasis.mp. or exp Metastasis/ | 947 |
| 8 | exp "Relapse (Disorders)"/ or recurrence.mp. | 19108 |
| 9 | 5 or 6 or 7 or 8 | 108315 |
| 10 | 2 or 3 or 4 | 427757 |
| 11 | 1 and 9 and 10 | 340 |

**Supplementary Table S5**

Quality assessment according to the Newcastle-Ottawa Scale for the studies subjected to meta-analysis.

| Study | Pub year | Selection | Comparability | Outcome |
| --- | --- | --- | --- | --- |
| Waston | 2005 | **** | ** | *** |
| Bredal | 2011 | **** | * | *** |
| Chen | 2016 | **** | ** | * |
| Desai | 2019 | **** | ** | * |
| Goodwin | 2004 | **** | * | * |
| Eskelinen | 2017 | **** | - | ** |
| Iglay | 2017 | **** | * | ** |
| Kanani | 2016 | **** | ** | ** |
| Liang | 2017 | **** | ** | *** |
| Onitilo | 2006 | **** | ** | *** |
| Phillips | 2008 | **** | ** | *** |
| Shim | 2019 | **** | ** | * |
| Vodermaier | 2014 | **** | * | ** |
| Groenvold | 2007 | **** | ** | *** |
| Hjerl | 2003 | **** | ** | ** |
| Graham | 2002 | **** | * | *** |
| Batty | 2016 | **** | ** | *** |


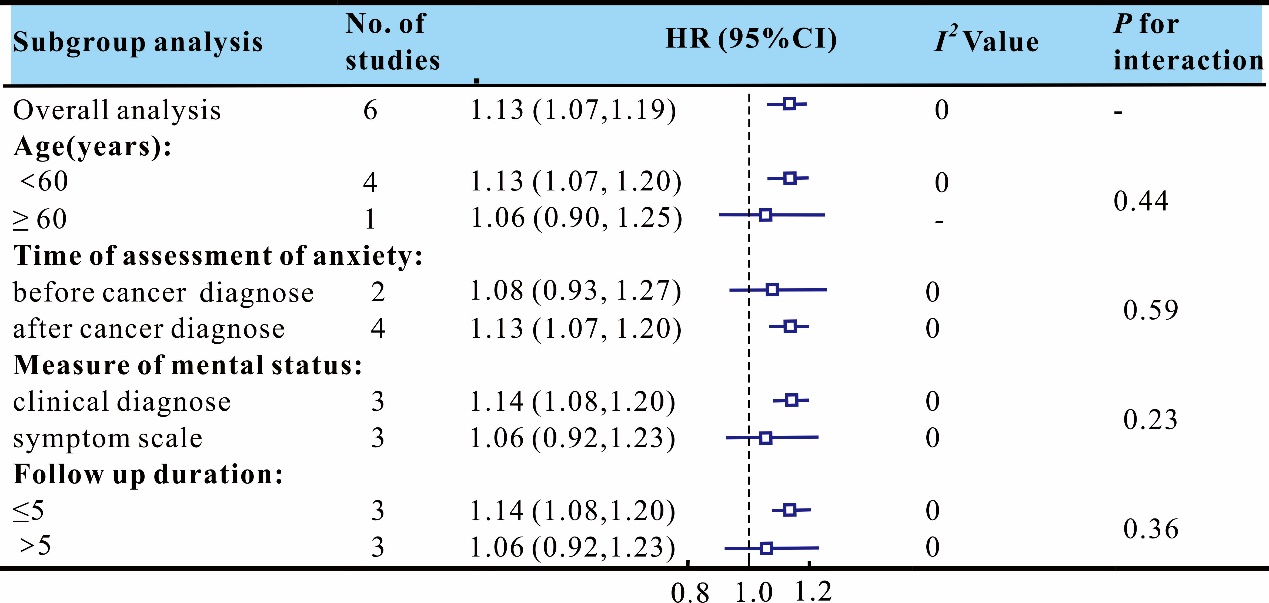


**Supplementary Figure S1** Subgroup analysis of the eﬀect of anxiety on all-cause mortality


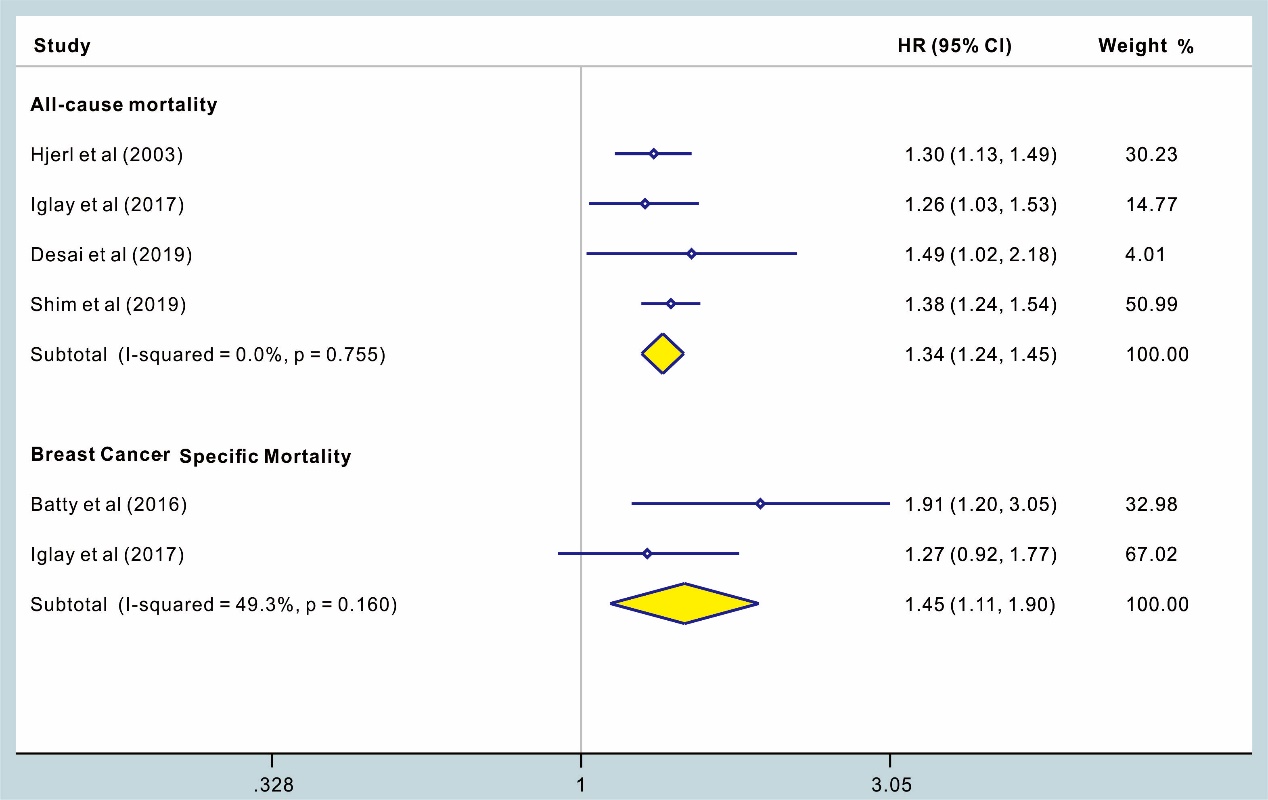


**Supplementary Figure S2** Forest plot illustrating the effects of depression and anxiety on all-cause mortality and cancer-specific mortality in patients with breast cancer.


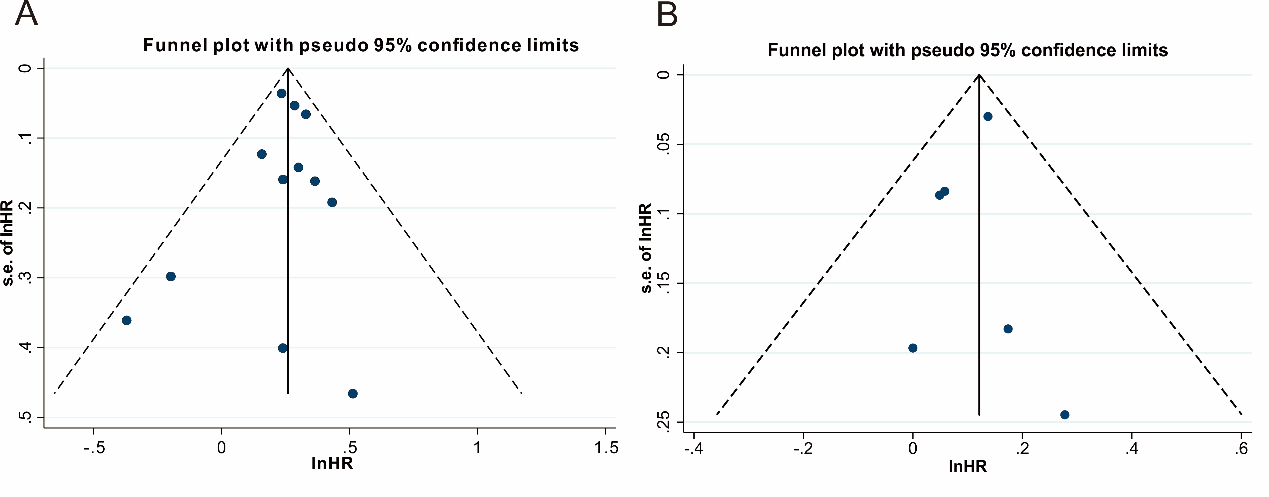


**Supplementary Figure S3** Funnel plots for the assessment of publication bias.

A: 12 studies related to depression. B: 6 studies related to anxiety. Each dot represents one included study. The vertical line indicates the pooled effect size; funnel indicates 95% confidence intervals.


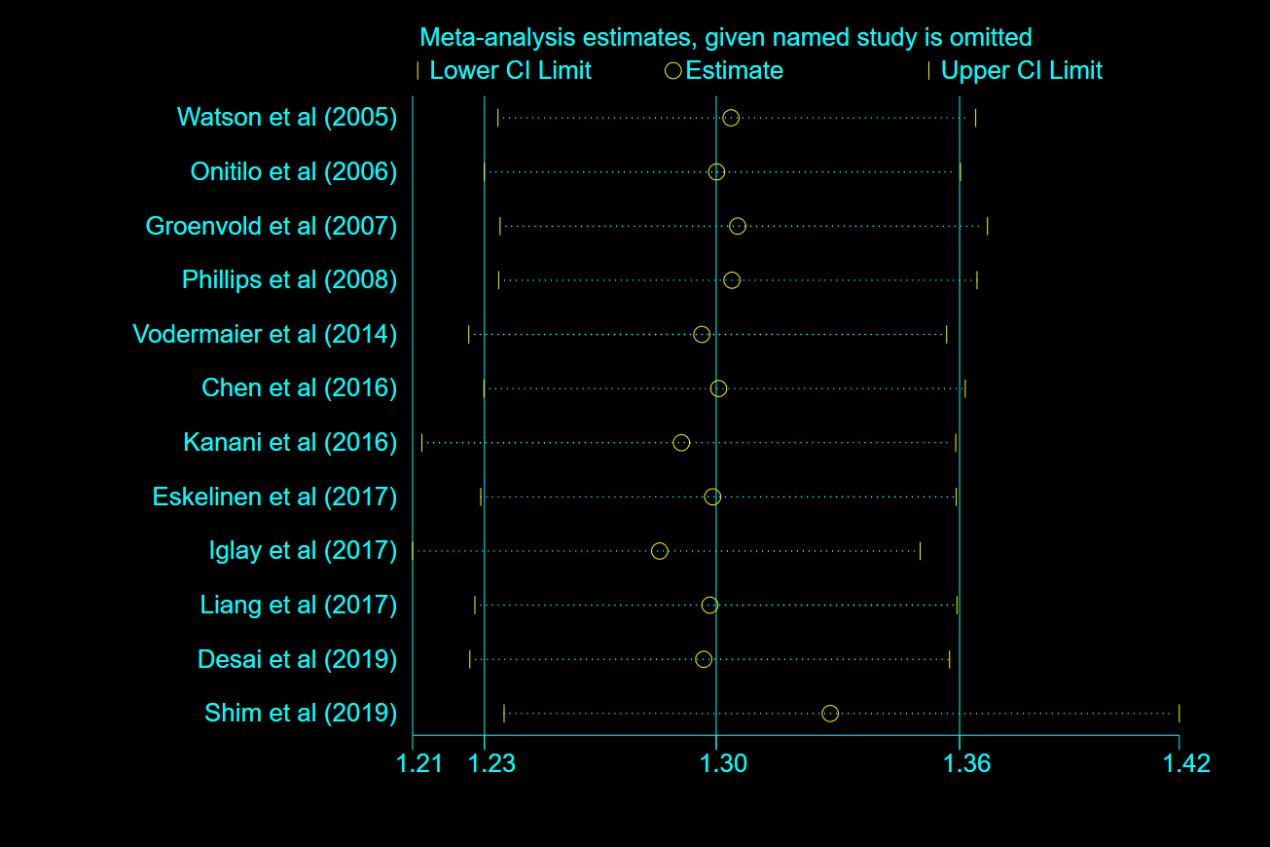


**Supplementary Figure S4** Sensitivity analysis. Estimates when omitting one study at each time.

**Supplementary** **Table S6**

Comparison between crude and adjusted association between mental disorder with mortality

| study | mental disorder | | crude HRs | | adjusted HRs |
| --- | --- | --- | --- | --- | --- |
| all-cause mortality | | | | | |
| Watson et al | | depression | | 0.72(0.36,1.47) | 0.69(0.34,1.4) |
| Onitilo et al | | depression | | 1.28(0.73,2.23) | 1.27(0.58,2.79) |
| Phillips et al | | depression | | 0.97(0.56,1.68) | 0.82(0.46-1.48) |
| Chen et al | | depression | | 1.354(0.999,1.834) | 1.271(0.93,1.737) |
| Kanani et al | | depression | | 1.96(1.77,2.17) | 1.33(1.2,1.48) |
| Liang et al | | depression | | 1.49(1.13,1.95) | 1.35(1.02,1.78) |
| Shim et al | | depression | | 1.349(1.257,1.447) | 1.264(1.178,1.357) |
| Watson et al | | anxiety | | 1.05(0.74,1.5) | 1.19(0.83,1.7) |
| Phillips et al | | anxiety | | 1.05(0.73,1.52) | 0.80(0.52,1.21) |
| Shim et al | | anxiety | | 1.253(1.182,1.328) | 1.147(1.081,1.216) |
| Shim et al | | depression and anxiety | | 1.518(1.366,1.689) | 1.383(1.243,1.538) |
| Recurrence-free survival | | | | | |
| Watson et al | | depression | | 0.73(0.37,1.42) | 0.70(0.36,1.39) |
| Phillips et al | | depression | | 1.15(0.72,1.85) | 0.82(0.49,1.38) |
| Bredal et al | | depression | | 2.68(1.24,5.79) | 1.79(0.95,4.06) |
| Chen et al | | depression | | 1.459(1.173,1.815) | 1.373(1.098.1.716) |
| Groenvold et al | | anxiety | | 1.01(0.72,1.42) | 1.19(1.02,1.39) |
| cancer-specified mortality | | | | | |
| Iglay et al | | depression and anxiety | | 1.48(0.95,2.3) | 1.09(0.86,1.39) |
